# Editorial: Women in science: Public mental health 2021

**DOI:** 10.3389/fpubh.2022.1005116

**Published:** 2022-08-26

**Authors:** Elisa H. Kozasa, Joanne Nicholson

**Affiliations:** ^1^Hospital Israelita Albert Einstein, São Paulo, SP, Brazil; ^2^Heller School for Social Policy and Management, Brandeis University, Waltham, MA, United States

**Keywords:** mental health, women, public health, women in science, COVID-19

Women continue to experience challenges in participation and inclusion in STEM fields (science, technology, engineering, and mathematics) (1). Women's participation is growing slowly. In a 2015–2018 UNESCO Institute for Statistics (UIS) survey, only 33.3% of people employed in research were women (2). Universities and policymakers in different regions of the world have been implementing strategies to reduce the gender gap in STEM (3). However, a low percentage of women fill higher and senior academic positions due, in part, to the lower number of papers published by women (4).

The goal of this Research Topic is to highlight female contributions to public mental health. The articles in this collection represent the work of 53 investigators from 8 countries, whose work focuses on the health and mental health of women as patients and providers; in low- and middle-income countries; dealing with the stresses of reproductive and obstetric issues, immigrant and employment status and, of course, the COVID pandemic.

In a systematic review, Prom et al. assessed the effectiveness of interventions that integrate perinatal mental health care into routine maternal care to improve infants and maternal outcomes in low- and middle-income countries, concluding that they can be effective. Gladstone et al. evaluated the effect of a cognitive behavior therapy, psycho-education approach on depression, anxiety and traumatic stress in women in Ethiopia with obstetric fistula, a serious complication in low-income countries.

Hynek et al. studied the ways in which mental disorder may affect women's income. Experiencing a mental disorder during the critical stage of establishing themselves in the workforce may negatively affect women's income as well as their future workforce participation, increasing dependency on social welfare services. Alshehri et al. demonstrated the validity and reliability of the Mental Health Literacy Scale—Arabic version with female university students, to facilitate the study of women's mental health during this vulnerable time period.

Articles regarding the impact of the COVID-19 pandemic on mental health, stress and burnout for women were submitted. A collaboration among Italian and Chinese investigators focused on promoting organizational and operational guidelines for the protection and wellbeing of healthcare workers, which were extensively disseminated in Italy (Scattoni et al.). The impact of the pandemic on women with fertility problems were evaluated by Biviá-Roig et al. The lifestyles of these women changed; anxiety and depression levels, and tobacco consumption increased during COVID-imposed confinement. A qualitative study to understand the effects of the COVID-19 pandemic on U.S. university students' mental health and how universities may best meet students' mental health needs was conducted by Kaur et al. Their findings suggest the value of improvements in communication and access to mental health resources. Zhou et al. examined the prevalence of burnout among Chinese female nurses during the controlled COVID-19 period. Burnout symptoms were associated with shorter job tenure, lower income, and working night shifts.

Women's mental health in relation to “normal” health challenges (e.g., reproductive health issues) and life circumstances (e.g., in the university setting) continues to be the focus of much-needed research, with particular concern for the development of valid, reliable measures/methods and effective intervention strategies, in low- and middle-income countries, as well as in those with greater resources. The COVID pandemic has exacerbated the stresses women face, at home, at school, and in the workplace, highlighting the need for enhanced communication and understanding of mental health, and improved access to effective care. The articles in this collection lay the groundwork for further study of these issues.

## Author contributions

All authors listed have made a substantial, direct, and intellectual contribution to the work and approved it for publication.

## Conflict of interest

The authors declare that the research was conducted in the absence of any commercial or financial relationships that could be construed as a potential conflict of interest.

## Publisher's note

All claims expressed in this article are solely those of the authors and do not necessarily represent those of their affiliated organizations, or those of the publisher, the editors and the reviewers. Any product that may be evaluated in this article, or claim that may be made by its manufacturer, is not guaranteed or endorsed by the publisher.
